# P86 Using the Australian National Antimicrobial Prescribing Survey (NAPS) portal to undertake an outpatient point prevalence survey (PPS)

**DOI:** 10.1093/jacamr/dlaf230.093

**Published:** 2025-12-04

**Authors:** Louise Dunsmure, Rachel Tan, Teig Parsons, Bee Yean Ng, Tanya Escayo, Hazera Haque, Nicola Jones

**Affiliations:** Department of Pharmacy, Oxford University Hospitals NHS Foundation Trust, Oxford, UK; Department of Pharmacy, Oxford University Hospitals NHS Foundation Trust, Oxford, UK; Department of Pharmacy, Oxford University Hospitals NHS Foundation Trust, Oxford, UK; Department of Pharmacy, Oxford University Hospitals NHS Foundation Trust, Oxford, UK; Department of Pharmacy, Oxford University Hospitals NHS Foundation Trust, Oxford, UK; Department of Pharmacy, Oxford University Hospitals NHS Foundation Trust, Oxford, UK; Department of Pharmacy, Oxford University Hospitals NHS Foundation Trust, Oxford, UK; Department of Infectious Diseases, Oxford University Hospitals NHS Foundation Trust, Oxford, UK

## Abstract

**Background:**

Annual point prevalence surveys (PPS) are key cornerstones in antimicrobial stewardship (AMS) helping to evaluate appropriateness of antimicrobial prescribing which can guide AMS interventions. PPS primarily focus on inpatients, but we undertook a PPS focusing on prescribing in the outpatient setting for patients in a tertiary hospital. NAPS is a web-based qualitative auditing platform that provides a standardized and validated tool to assist hospitals in assessing the appropriateness of antimicrobial prescribing practices.

**Objectives:**

To use NAPS to assess appropriateness of antimicrobial prescribing in an outpatient setting and evaluate the NAPS tool usefulness in a UK setting.

**Methods:**

Permission to use the platform was given by NAPS on a pilot basis. Over a 5 day period all outpatients at one hospital site prescribed an antimicrobial were extracted using Cerner electronic prescribing system. Further detail including indication, appropriateness and compliance to guideline was reviewed, anonymized and uploaded by the trained individuals to NAPS.

**Results:**

88 patients were prescribed antimicrobials equating to 100 prescriptions. Three most common specialities were the Emergency Department (ED) (33% (*n*=33) of antimicrobials prescribed), Maxillofacial/ENT (21%) and Ophthalmology (14%). The most common indications for antimicrobials were genitourinary infections 23% of prescriptions (*n*=23), skin and soft tissue infections in 21% and respiratory infections in 17% of prescriptions. Overall compliance with guidelines was achieved in 89% of prescriptions and 72% were deemed appropriate (optimal or adequate). The most commonly prescribed antimicrobial was co-amoxiclav (18%) followed by amoxicillin (10%) and nitrofurantoin (8%). Inappropriate prescriptions were more prevalent for co-amoxiclav (22% *n*=6/27) and amoxicillin (15%). The most common reasons for inappropriate prescription were incorrect (prolonged) duration (41% *n*=11/27) and PPS assessor concluded that the antimicrobial was not indicated (30% *n*=8/27).

**Conclusions:**

Using NAPS, we have identified that ED had the largest number of outpatient antimicrobials, and that genitourinary infections are the leading indication for antimicrobial prescribing. Co-amoxiclav is the main antibiotic being prescribed and that appropriateness of this and other antibiotics are below the 90% target. Consequently, targeted AMS efforts can be designed towards improving these outcomes. The result of the PPS is also fed back to respective team to inform prescribing and has been used as part of teaching sessions to encourage teams to take ownership of their own data and influence prescribing practice. A recognized limitation of PPSs is that the assessment of appropriateness is subjective and to minimize this only trained staff undertake the PPS work. We have found NAPS to be user friendly and reliable. Reports are produced immediately on completion of the survey to allow timely feedback. We would recommend NAPS as a useful tool in performing PPS and as a potential way to benchmark NHS trusts across the UK.

